# ASAP1 Promotes Epithelial to Mesenchymal Transition by Activating the TGFβ Pathway in Papillary Thyroid Cancer

**DOI:** 10.1002/cam4.71075

**Published:** 2025-07-31

**Authors:** Shiji Song, Zixing Leng, Xinxin Zhao, Ziping Liu, Yongshuai Li, Wenjing Zhang, Junming Yue, Yuxia Fan

**Affiliations:** ^1^ Department of Thyroid Surgery The First Affiliated Hospital, Zhengzhou University Zhengzhou Henan People's Republic of China; ^2^ Department of Gynecology and Obstetrics The Third Affiliated Hospital, Zhengzhou University Zhengzhou Henan People's Republic of China; ^3^ Department of Pathology and Laboratory Medicine College of Medicine, University of Tennessee Health Science Center Memphis Tennessee USA; ^4^ Center for Cancer Research University of Tennessee Health Science Center Memphis Tennessee USA

**Keywords:** ASAP1, EMT, interaction, invasion, PTC, TGFβ

## Abstract

**Background:**

Papillary thyroid cancer (PTC) is the most common type of thyroid malignancy. While the prognosis of PTC is generally favorable, some cases exhibit aggressive behavior, leading to metastasis and recurrence. ASAP1 (ArfGAP with SH3, ankyrin repeats, and PH domain 1), an ADP‐ribosylation factor GTPase‐activating protein, has been implicated in tumor metastasis. However, its role in PTC remains unclear.

**Methods:**

ASAP1 expression in PTC was evaluated using TCGA and GEO databases. Studies in PTC cell lines (MDA‐T32 and MDA‐T85) included lentiviral‐mediated knockdown and overexpression of ASAP1 to assess effects on epithelial–mesenchymal transition (EMT) marker expression, cell proliferation, and invasive capacity. TGFβ pathway activity was examined by p‐SMAD2 Western blotting and luciferase reporter assays. ASAP1‐SMAD2/3 interactions were analyzed using co‐immunoprecipitation (CO‐IP) and immunofluorescence.

**Results:**

ASAP1 was aberrantly upregulated in PTC. Lentiviral knockdown of ASAP1 in PTC cells suppressed the EMT process. Reduced ASAP1 expression also inhibited cell survival, proliferation, migration, invasion, and the expression of p‐SMAD2 in the TGFβ pathway in PTC cells. Conversely, ASAP1 overexpression reversed these effects. Mechanistically, ASAP1 interacts with the SMAD2/3 complex, forming a positive feedback loop with TGFβ signaling that promotes EMT and cell invasiveness in PTC cells, which suggests its potential role in PTC metastasis.

**Conclusions:**

These findings suggest that targeting ASAP1 may offer a novel therapeutic strategy to limit PTC metastasis by suppressing EMT and attenuating the TGFβ pathway.

## 1 Introduction

Thyroid cancer (TC) is the most common endocrine malignancy worldwide, with a steadily rising global incidence over the past several decades—particularly among women [1]. Epidemiological studies have identified radiation exposure, abnormal iodine intake, family history, and certain genetic mutations (such as BRAFV600E) as major risk factors for TC [2]. Additionally, some studies suggest that obesity, metabolic syndrome, sex, and hormone levels may also contribute to its development [3, 4]. Papillary thyroid cancer (PTC) represents the predominant histological subtype, accounting for 80%–85% of all thyroid cancers [5, 6]. Although PTC generally has a favorable prognosis, certain cases exhibit invasive behavior, leading to metastasis and recurrence [7]. Treatments for patients with metastatic PTC remain limited [8]. Radioactive iodine (RAI) therapy is commonly used to treat metastatic PTC; however, its effectiveness is significantly reduced in patients who develop RAI‐refractory PTC [9, 10].

The underlying molecular mechanisms driving metastatic PTC remain incompletely understood. Accumulated evidence indicates that invasive PTC cells undergo epithelial‐to‐mesenchymal transition (EMT) and activate multiple signaling pathways including the TGFβ, NF‐kB, and integrin pathways [11]. EMT is characterized by significant changes in cellular phenotype, such as the loss of epithelial traits (e.g., E‐cadherin) and the acquisition of mesenchymal characteristics (e.g., N‐cadherin and Vimentin) [12]. This phenotypic shift enables tumor cells to detach from the primary site, invade surrounding tissues, and eventually disseminate to distant organs through lymphatic or vascular pathways [13]. Therefore, identifying the key factors involved in EMT is crucial for elucidating the mechanisms underlying PTC metastasis and offers new avenues for developing targeted therapeutic strategies, particularly for patients with RAI‐refractory PTC.

ASAP1, also known as ArfGAP with SH3, ankyrin repeat, and PH domains 1 [14], is overexpressed in various cancers [15, 16, 17, 18, 19, 20, 21, 22, 23, 24]. Notably, ASAP1 plays a critical role in the EMT process across different cancers and has been shown to promote EMT in ovarian, gastric, and breast cancers [21, 25, 26]. Our previous research indicates that ASAP1 contributes to the autophagy process by regulating the mTOR pathway [20]. However, the specific role of ASAP1 in PTC requires further investigation.

This study aims to systematically investigate the role of ASAP1 in EMT, migration, and invasion in PTC cells, as well as its regulatory interplay with the TGFβ pathway. The findings will enhance our understanding of the molecular mechanisms underlying PTC progression and offer a theoretical basis for developing novel targeted therapies.

## 2 Materials and Methods

### 2.1 Bioinformatics Analysis

Transcriptomic data for PTC were obtained from the Cancer Genome Atlas (TCGA) database (https://portal.gdc.cancer.gov/). The TCGA dataset for thyroid cancer (THCA) comprises 500 PTC tissues (PTCs) and 57 normal tissues. Additionally, three independent datasets were retrieved from the Gene Expression Omnibus (GEO) database (http://www.ncbi.nlm.nih.gov/geo): GSE60542 includes 30 normal tissues and 33 PTCs, GSE58545 includes 18 normal tissues and 27 PTCs, and GSE33630 includes 45 normal tissues, 49 PTCs, and 11 anaplastic thyroid cancer tissues (ATCs). The transcriptomic data from normal tissues, PTCs, and ATCs in these databases were used for bioinformatic analysis.

Patients in the THCA dataset were stratified into high and low ASAP1 expression groups based on the median expression value of ASAP1. Differentially expressed genes (DEGs) between the two groups were identified using the “Deseq2” package, with significance defined at *p* < 0.05 and |log_2_ fold change (FC)| ≥ 1. Using the “clusterProfiler” package in R (version 4.2.1), we performed Gene Ontology (GO) and The Kyoto Encyclopedia of Genes and Genomes (KEGG) analyses on the DEGs [27, 28]. The correlation between ASAP1 and other genes in the THCA dataset was analyzed using the TIMER database (http://TIMER.cistrome.org/).

### 2.2 Cell Culture

PTC cell lines (MDA‐T32 and MDA‐T85) were obtained from the ATCC (Manassas, VA) and cultured in RPMI‐1640 medium supplemented with 10% FBS (Corning, Corning, NY), 100 U/mL penicillin, and 100 μg/mL streptomycin (Invitrogen, Carlsbad, CA). Cells were maintained at 37°C in an incubator with 5% CO_2_. Recombinant Human TGF‐beta1 (carrier‐free) (TGFβ1) was purchased from BioLegend (Cat. No. 781804, Biolegend, San Diego, CA), and its working concentration in culture medium was 10 ng/mL. The TGFβR1 inhibitor SB431542 was purchased from Reagents Direct (Cat. No. 21‐A94, Reagents Direct, Encinitas, CA), and its working concentration was 20 μM in culture medium.

### 2.3 Lentiviral Vector Production

Lentiviral vector for ASAP1 overexpression or shRNA vectors were packaged in 293FT cells as described previously [29]. Stable cell lines were established by transducing the MDA‐T32 and MDA‐T85 cells with the lentiviral ASAP1 or control vector (shRNA against EGFP), and selected with 2 μg/mL puromycin (Cat. No. ant‐pr‐1, InvivoGen, San Diego, CA).

### 2.4 Cell Migration Assay

Transwell migration assays were performed using cells in the logarithmic growth phase. Briefly, MDA‐T32 or MDA‐T85 cells (3 × 10^4^ cells/well) were suspended in 300 μL of serum‐free medium and added to the upper chamber of the Transwell inserts (BD Falcon, San Jose, CA). In the lower chamber, 700 μL of medium supplemented with 10% FBS was added. After a 24‐h incubation, nonmigrated cells in the upper chamber were removed, and cells that had migrated to the underside of the membrane were fixed with methanol and stained with 0.1% crystal violet solution. Images were captured at 200× magnification, and migrated cells were counted in at least three randomly selected fields using ImageJ software.

### 2.5 Cell Invasion Assay

Matrigel gel (Cat. No. 354248, Corning, Corning, NY) was diluted 1:8 in serum‐free medium. Fifty microliters of the diluted Matrigel was added to the upper chamber of Transwell inserts and incubated at 37°C for 1 h. MDA‐T32 or MDA‐T85 cells (2 × 10^5^ cells/well) were then suspended in 300 μL of serum‐free RPMI 1640 medium and seeded in the upper chamber. RPMI 1640 medium supplemented with 10% FBS was used as a chemoattractant in the lower chamber. After a 48‐h incubation, invaded cells were fixed and stained with hematoxylin and eosin (H&E) and counted as described in the cell migration assay.

### 2.6 Cell Proliferation Assay by Incucyte

MDA‐T32 or MDA‐T85 cells (2.5 × 10^3^ cells/well) were seeded in 96‐well plates using 200 μL of culture medium supplemented with 10% FBS. The plates were incubated in an IncuCyte Live Cell Analysis System (Sartorius, Göttingen, Germany) at 37°C with 5% CO_2_. Cell proliferation was monitored in real time using phase‐contrast imaging, and cellular confluency in each well was recorded at 4‐h intervals for 96 h.

### 2.7 Cell Clonogenic Assay

MDA‐T32 or MDA‐T85 cells (300 cells/well) were plated in 6‐well plates and cultured for 10 days, then stained with 0.1% Crystal Violet. Cell colonies were counted using ImageJ software, and then statistical analysis was performed on three different wells.

### 2.8 Western Blot (WB)

PTC cells were collected in RIPA buffer (Thermo Scientific, Rockford, IL) containing 1% Halt proteinase inhibitor cocktail (Thermo Scientific, Rockford, IL). Equal amounts of protein (40 μg/lane) were loaded onto 10% SDS‐PAGE gels and transferred onto 0.45 μm PVDF membranes (Millipore Corp, Billerica, MA). The membranes were blocked with 5% nonfat milk for 1 h and incubated with primary antibodies against ASAP1 (1:1000, #sc‐374,410, Santa Cruz Biotechnology, Dallas, TX), N‐cadherin (1:1000, #13116 s), Vimentin (1:1000, #5741 s), SMAD2/3 (1:1000, #8685 s), phosphor‐SMAD2 (p‐SMAD2) (1:1000, #AB3849‐I, MilliporeSigma, Rockville, MD), Cytokeratin 7 (1:6000, #ab181598, Abcam, Cambridge, MA), and GAPDH (1:4000, #sc‐47,724, Santa Cruz Biotechnology, Dallas, TX) for 12 h at 4°C. After washing three times with PBST for 5 min each, membranes were incubated with secondary antibody for 1 h at room temperature. The membranes were incubated with enhanced chemiluminescence (ECL) solution for 2 min and exposed to X‐ray film. Band intensity in the WB was measured using ImageJ software.

### 2.9 Luciferase Reporter Assay

ASAP1 control and knockdown MDA‐T32 or MDA‐T85 cells were transduced using a lentiviral vector pGF‐SMAD2/3/4‐mCMV‐luciferase‐EF1a‐puro (System Biosciences, CA) containing the firefly luciferase reporter gene under the control of SMAD2/3/4 transcriptional response elements. Following transduction, cells were treated with either vehicle or 10 ng/mL TGFβ1 for 24 h. Luciferase activity was measured using the Dual‐Luciferase Reporter Assay System (Promega, Madison, WI, USA) to quantify SMAD pathway activation, and results were normalized to cell numbers.

### 2.10 Reverse Transcription Quantitative PCR (RT–qPCR)

Following the manufacturer's protocol, total RNA was isolated from PTC cells using TRIzol reagent (Cat. No. 15596026, Ambion, Austin, TX). Five micrograms of total RNA was reverse‐transcribed into cDNA using the Maxima First Strand cDNA Synthesis Kit for RT‐qPCR (Cat. No. K1641, Thermo Scientific, Rockford, IL) in a 20 μL reaction. For qPCR analysis, the 10 μL RT‐qPCR reaction consisted of 5 μL PowerUp SYBR Green Master Mix (Thermo Scientific, Rockford, IL), 0.3 μL forward primer, 0.3 μL reverse primer, and 4.4 μL of 40 × diluted cDNA. The qPCR cycling conditions were as follows: initial denaturation (95°C for 2 min), followed by 40 cycles of denaturation at 95°C for 15 s and annealing/extension at 60°C for 1 min. A melt curve analysis was performed at the end of the PCR reaction. Primer sequences used in this study are listed in Table 1. The relative expression was calculated using the 2^−∆∆Ct^ method, with β‐actin as the endogenous control.

**TABLE 1:** Primer sequences used in RT‐qPCR.

Primer name	Sequence (5′ → 3′)
ASAP1 forward	TAGAACAGCCCTTCAGAAAGTGA
ASAP1 reverse	CGGGGTTGTCTCGACTTAAAAA
β‐actin forward	AGCACCATGAAGATCAAGATCATT
β‐actin reverse	CGGACTCATCGTACTCCTGCTT

### 2.11 Co‐Immunoprecipitation Assay (Co‐IP)

Cells were lysed on ice for 25 min using lysis buffer containing 25 mM Tris, 150 mM NaCl, 1 mM EDTA, 1% NP‐40, and 5% glycerol (pH 7.4). A 40‐μg aliquot of protein lysate was retained as an input control. Dynabeads M‐280 sheep anti‐mouse/rabbit IgG (Thermo Fisher Scientific, Rockford, IL) were preincubated with anti‐ASAP1 (1 μg, #sc‐374,410, Santa Cruz Biotechnology) or anti‐SMAD2/3 (0.5 μg, #8685 s, Cell Signaling Technology) and control mouse/rabbit IgG (0.5 μg, #sc‐2025/sc‐2027, Santa Cruz Biotechnology) for 30 min at room temperature. The beads were then washed three times with PBS containing 2 mM EDTA and 0.1% bovine serum albumin (BSA). Subsequently, 4 mg of protein lysate was added to the beads and incubated for an additional 30 min. After washing three times, 20 μL of loading buffer was added. The samples were then boiled, and WB analysis was performed.

### 2.12 Protein Docking

The 3D structure files of ASAP1 SH3 domain (PDB ID: 2RQT) and SMAD2 (PDB ID: 6H3R) were retrieved in PDB format from the Protein Data Bank (PDB) (https://www.rcsb.org/). Subsequently, their potential interactions were predicted using the HDOCK server [30] (http://hdock.phys.hust.edu.cn/) and the results were visualized using PyMOL (Version 3.1.0) [31] and LigPlot^+^ (Version 2.2.9).

### 2.13 Immunofluorescence Assay (IF)

ASAP1 control and knockdown MDA‐T85 cells (2 × 10^4^ cells/well) were seeded into 24‐well plates. Following cell adherence, the cells were cultured in serum‐free medium and treated with either vehicle or TGFβ1 (10 ng/mL) for 24 h. The cells were then fixed with methanol for 10 min, permeabilized with 0.1% Triton X‐100 for 15 min, and blocked with 5% BSA in PBS for 1 h. After washing with PBS three times for 5 min each, primary antibodies against ASAP1 (1:50, #sc‐374,410, Santa Cruz Biotechnology), Vimentin (1:100, #5741 s, Cell Signaling Technology), Cytokeratin 7 (1:800, #ab181598, Abcam), and SMAD2/3 (1:400, #8685 s, Cell Signaling Technology) were incubated overnight at 4°C. The cells were then incubated with secondary antibodies: goat anti‐mouse/rabbit conjugated with Alexa Fluor 568/488 (1:500, Invitrogen, Carlsbad, CA) at room temperature for 1 h. Cell nuclei were counterstained with DAPI (Vector Laboratories Inc). Images were captured using a fluorescence microscope (Nikon, San Diego, CA) at 400× magnification.

### 2.14 Statistical Analysis

All data were analyzed using GraphPad Prism 9 and tested for normality and homogeneity of variance prior to analysis. For comparisons between two groups with equal variances, Student's t‐test was used. One‐way ANOVA was applied for comparisons among multiple groups, while two‐way ANOVA was used to analyze the effects of multiple factors and their interactions, followed by Dunnett's post hoc test. All data were normally distributed and are presented as mean ± SD. *p* < 0.05 was considered statistically significant.

## 3 Results

### 3.1 ASAP1 Is Upregulated in PTC Patients and Is Associated With ECM Remodeling and the TGFβ Signaling Pathway

**FIGURE 1:**
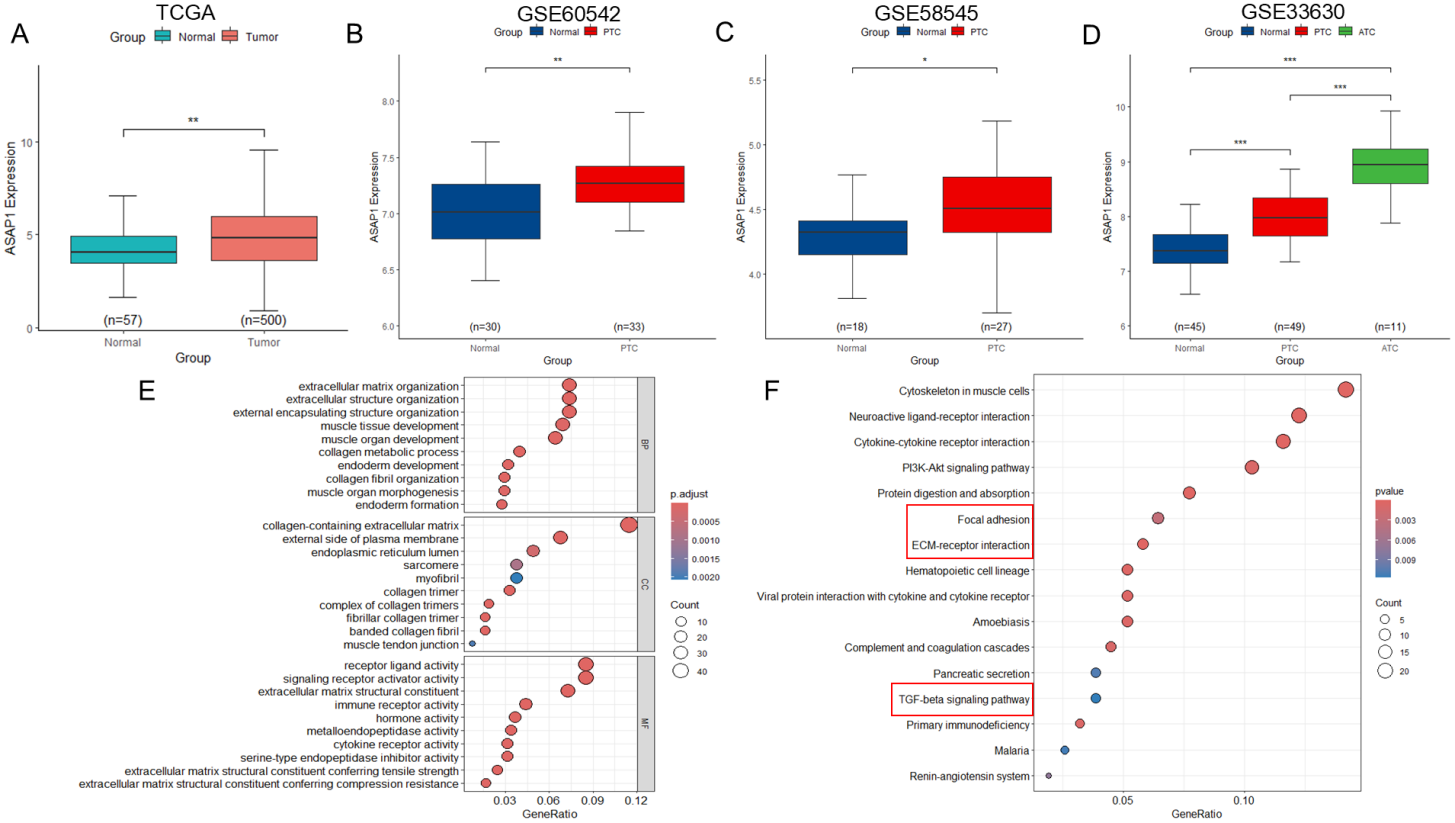
Expression and enrichment analysis of ASAP1 in PTC. (A, B, C, D) ASAP1 expression is elevated in PTC compared to normal thyroid tissues in the THCA and GEO datasets. Additionally, ASAP1 expression is higher in the more aggressive ATC compared to PTC. (E) GO enrichment analysis of DEGs in the high and low ASAP1 expression groups in the THCA dataset. (F) KEGG enrichment analysis of DEGs.

Our previous study, using immunohistochemistry, demonstrated elevated ASAP1 expression in PTC tissues [20]. To further validate this finding and explore the expression pattern of ASAP1, we analyzed data from multiple publicly available databases, including TCGA and GEO (GSE60542, GSE58545, GSE33630). Consistent with our previous findings, ASAP1 expression was significantly upregulated in PTC compared to normal thyroid tissues across all datasets analyzed (*p* < 0.05). Notably, ASAP1 expression was further increased in ATC, a more aggressive subtype (Figure 1A–D). To further investigate the biological functions and pathways potentially regulated by ASAP1 in PTC, we performed GO and KEGG enrichment analyses. Differentially expressed genes (DEGs) between high and low ASAP1 expression groups within the TCGA THCA dataset were identified using a |log2FC| > 1, *p* < 0.05 ‐cutoff. GO analysis revealed significant enrichment of DEGs in biological processes (BP) associated with ECM organization, including extracellular structure and encapsulating structure organization. In the cellular component (CC) category, DEGs were enriched in ECM‐related structures such as collagen‐containing ECM and the external plasma membrane. Molecular function (MF) analysis identified receptor ligand activity and ECM structural constituents as key features (Figure 1E). KEGG pathway analysis further revealed significant enrichment in cancer‐related pathways, including the PI3K‐Akt, focal adhesion, ECM‐receptor interaction, and TGF‐β signaling pathway (Figure 1F). These findings suggest that ASAP1 may play a critical role in PTC invasion by remodeling the ECM and driving key oncogenic pathways.

### 3.2 Lentiviral‐Mediated ASAP1 Knockdown Inhibits EMT in PTC Cells

**FIGURE 2:**
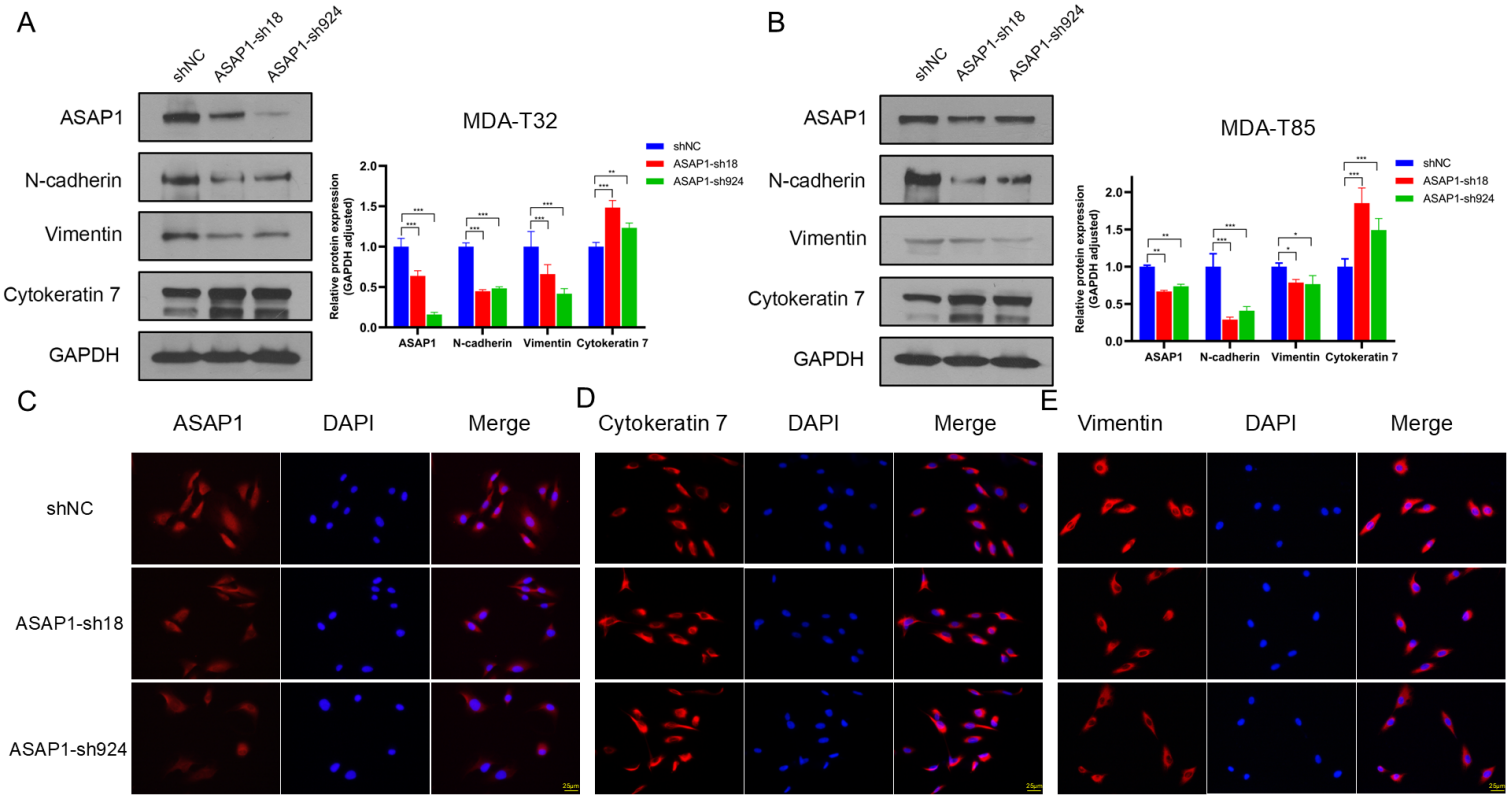
Knocking down ASAP1 inhibits EMT in PTC cells. (A) WB and quantitative analysis of ASAP1 and EMT markers in MDA‐T32 cells. (B) WB and quantitative analysis of ASAP1 and EMT markers in MDA‐T85 cells (*p < 0.05, **p < 0.01, ***p < 0.001). (C, D, E) IF showing ASAP1 (red, C); Cytokeratin 7 (red, D); Vimentin (red, E) expression in the control and ASAP1 knockdown groups of MDA‐T85 cells.

Given the elevated expression of ASAP1 in PTC, we hypothesized that ASAP1 promotes tumor progression in PTC cells. To test this hypothesis, we selected the PTC cell lines MDA‐T32 and MDA‐T85, the latter derived from metastatic cervical lymph nodes and exhibiting higher ASAP1 expression levels (Figure S1). Then, we used a lentiviral vector to knock down ASAP1 in both cell lines and investigated its impact on the expression of EMT markers in PTC cells. As shown in Figure 2A,B, knocking down ASAP1 increased the expression of the epithelial marker Cytokeratin 7, while decreasing the expression of mesenchymal markers N‐cadherin and Vimentin in both MDA‐T32 and MDA‐T85 cells. IF staining further confirmed the changes in ASAP1, Cytokeratin 7, and Vimentin expression in MDA‐T85 cells (Figure 2C–E). These results demonstrated that knockdown of ASAP1 inhibits EMT in PTC MDA‐T32 and MDA‐T85 cells.

### 3.3 Knockdown of ASAP1 Inhibits Cell Survival, Proliferation, Migration, and Invasion in PTC Cells

**FIGURE 3:**
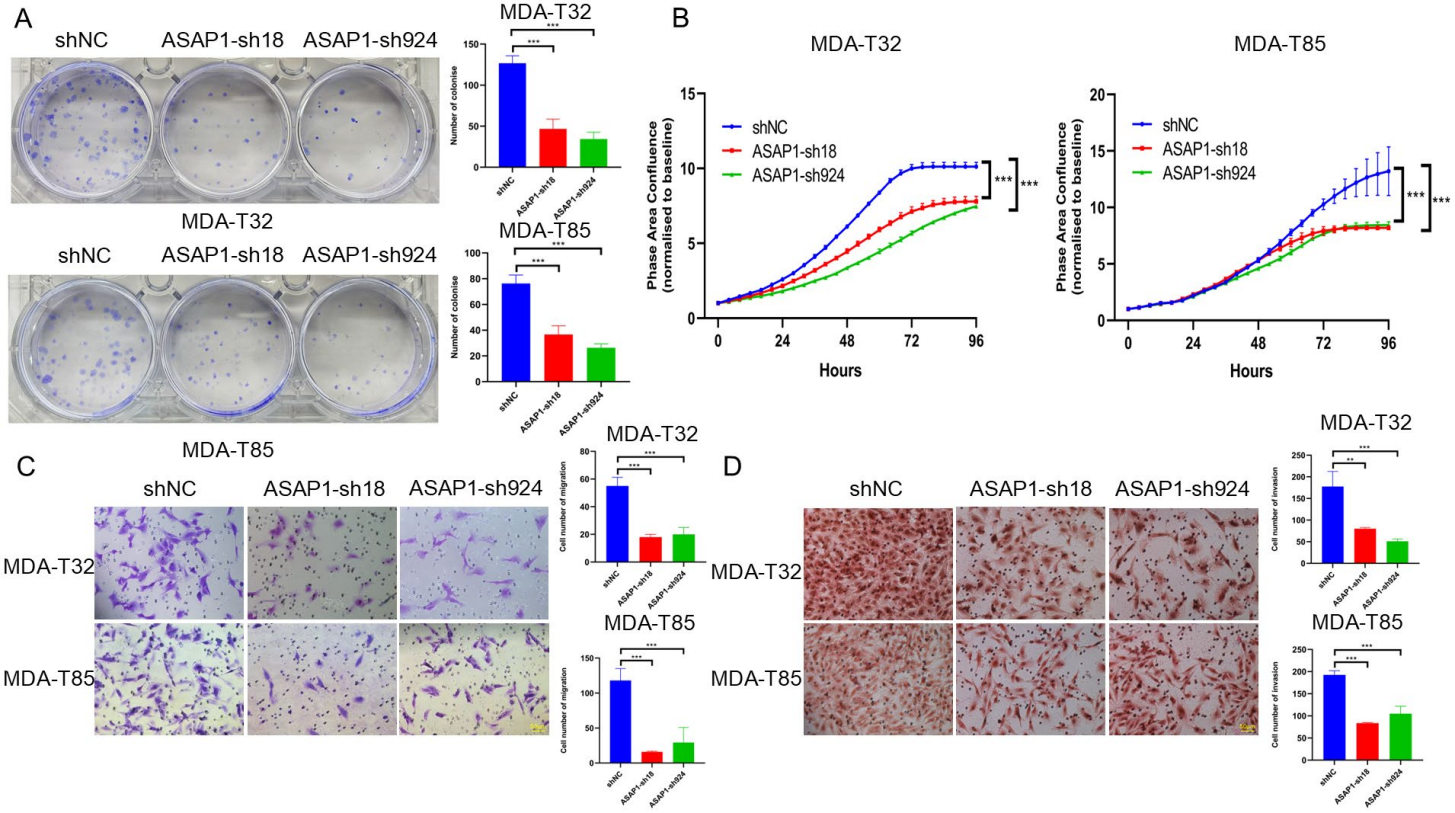
Knocking down ASAP1 inhibits colony formation, proliferation, migration, and invasion in PTC cells. (A) Comparison of cell colonies formation between control and ASAP1 knockdown groups in both MDA‐T32 and MDA‐T85 cell lines, stained with crystal violet. Right panels show the quantitative analysis. (B) The proliferation capacity of control and ASAP1 knockdown groups in MDA‐T32 and MDA‐T85 cells was measured using Incucyte system. (C) Transwell migration assay in MDA‐T32 and MDA‐T85 cells with control and ASAP1 knockdown groups, with migrated cells stained and counted using crystal violet. Right panels show the quantitative analysis. (D) Invasion assay using Matrigel‐coated transwell chambers in MDA‐T32 and MDA‐T85 cells with control and ASAP1 knockdown groups, with invaded cells stained and counted using H&E. Right panels show the quantitative analysis. (*p < 0.05, **p < 0.01, ***p < 0.001).

Given the elevated expression of ASAP1 in PTC cells and its role in promoting EMT, we hypothesized that ASAP1 contributes to tumor cell growth and invasiveness. Therefore, we investigated the effects of ASAP1 knockdown on cell survival, proliferation, migration, and invasion in MDA‐T32 and MDA‐T85 cells. Cell survival was assessed using colony formation assays. Compared with the control group, ASAP1 knockdown significantly reduced colony formation in both MDA‐T32 and MDA‐T85 cells (Figure 3A). To determine whether ASAP1 plays a role in cell proliferation, we monitored cell confluence every 4 h using the Incucyte system. Knockdown of ASAP1 significantly inhibited the proliferation of both MDA‐T32 and MDA‐T85 cells (Figure 3B). Furthermore, ASAP1 knockdown significantly suppressed cell migration and invasion in both MDA‐T32 and MDA‐T85 cells (Figure 3C,D).

### 3.4 Overexpression of ASAP1 Reverses the Effects of ASAP1 Knockdown on EMT and Cell Functions

**FIGURE 4:**
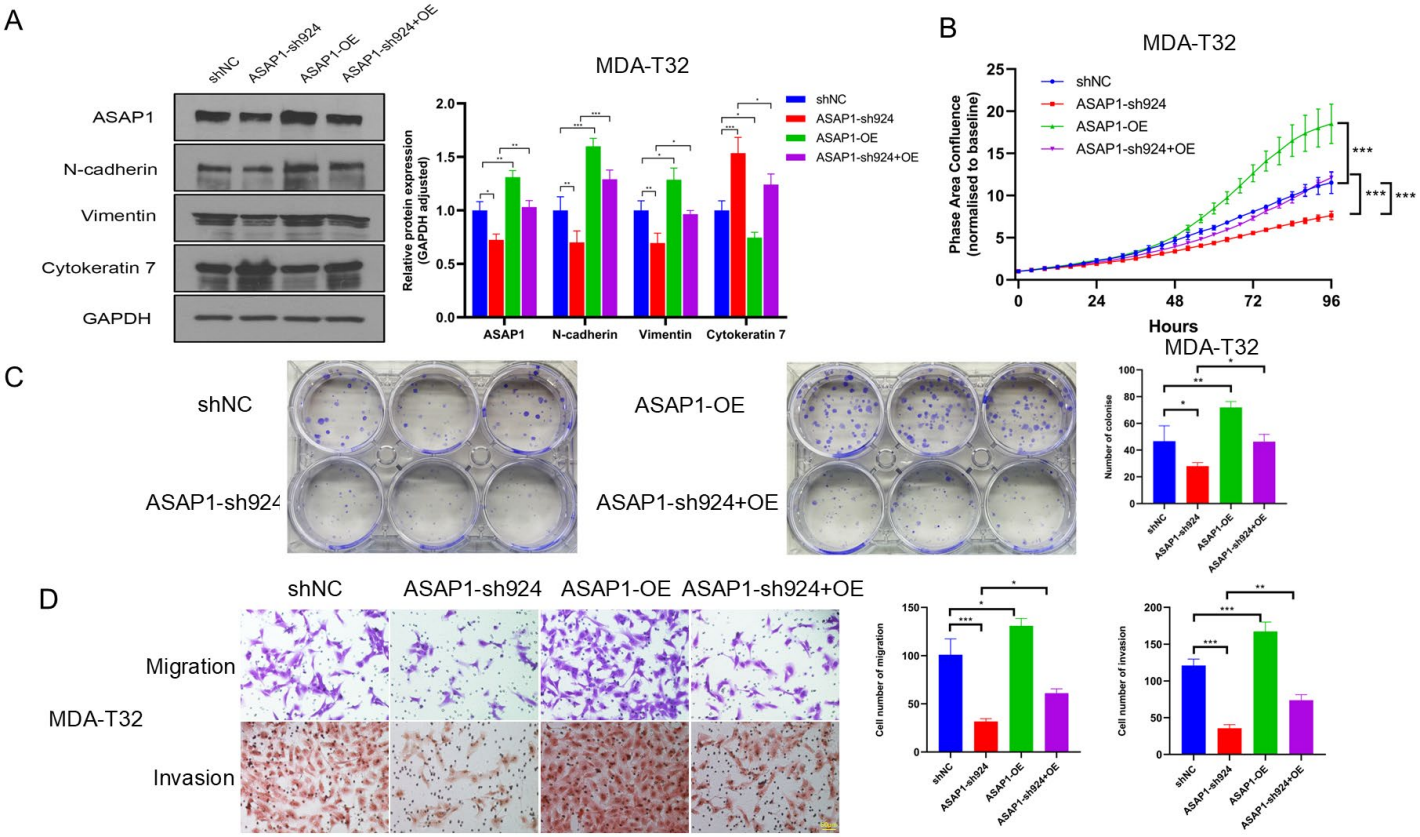
Overexpression of ASAP1 reverses EMT and the suppression of cellular functions caused by ASAP1 knockdown in MDA‐T32 cells. (A) WB and quantitative analysis of ASAP1 and EMT markers were performed in MDA‐T32 cells across control, ASAP1 knockdown, ASAP1 overexpression, and combined knockdown/overexpression groups. (B) Cell proliferation measurements in the four groups using IncuCyte system. (C) Colony formation among the four groups of MDA‐T32 cells was compared using crystal violet staining. Right panel shows the quantitative analysis. (D) Transwell migration and invasion assays were performed in the four groups of cells, with migrated cells stained and counted using crystal violet, while invaded cells were stained and counted using HE. Right panel shows the quantitative analysis. (*p < 0.05, **p < 0.01, ***p < 0.001).

To further define the impact of ASAP1 expression on PTC cellular function, we selected MDA‐T32 cells with relatively low ASAP1 expression compared to MDA‐T85 cells to perform a rescue assay by restoring ASAP1 expression in ASAP1 knockdown (sh924) MDA‐T32 cells. ASAP1 knockdown (sh924) cells were established by targeting the 3′UTR of the ASAP1 gene; thus, this will not affect the exogenously overexpressed ASAP1 in MDA‐T32 knockdown cells. Compared to the control group, ASAP1 overexpression increased the expression of mesenchymal markers N‐cadherin and Vimentin while reducing the expression of the epithelial marker Cytokeratin 7, thereby reversing the EMT inhibition observed in ASAP1 knockdown cells (Figure 4A). Functionally, ASAP1 overexpression significantly enhanced proliferation, colony formation, migration, and invasion in MDA‐T32 cells (Figure 4B–D), effectively rescuing the inhibitory effects of ASAP1 knockdown.

### 3.5 ASAP1 Modulates TGFβ Pathway in PTC Cells

**FIGURE 5:**
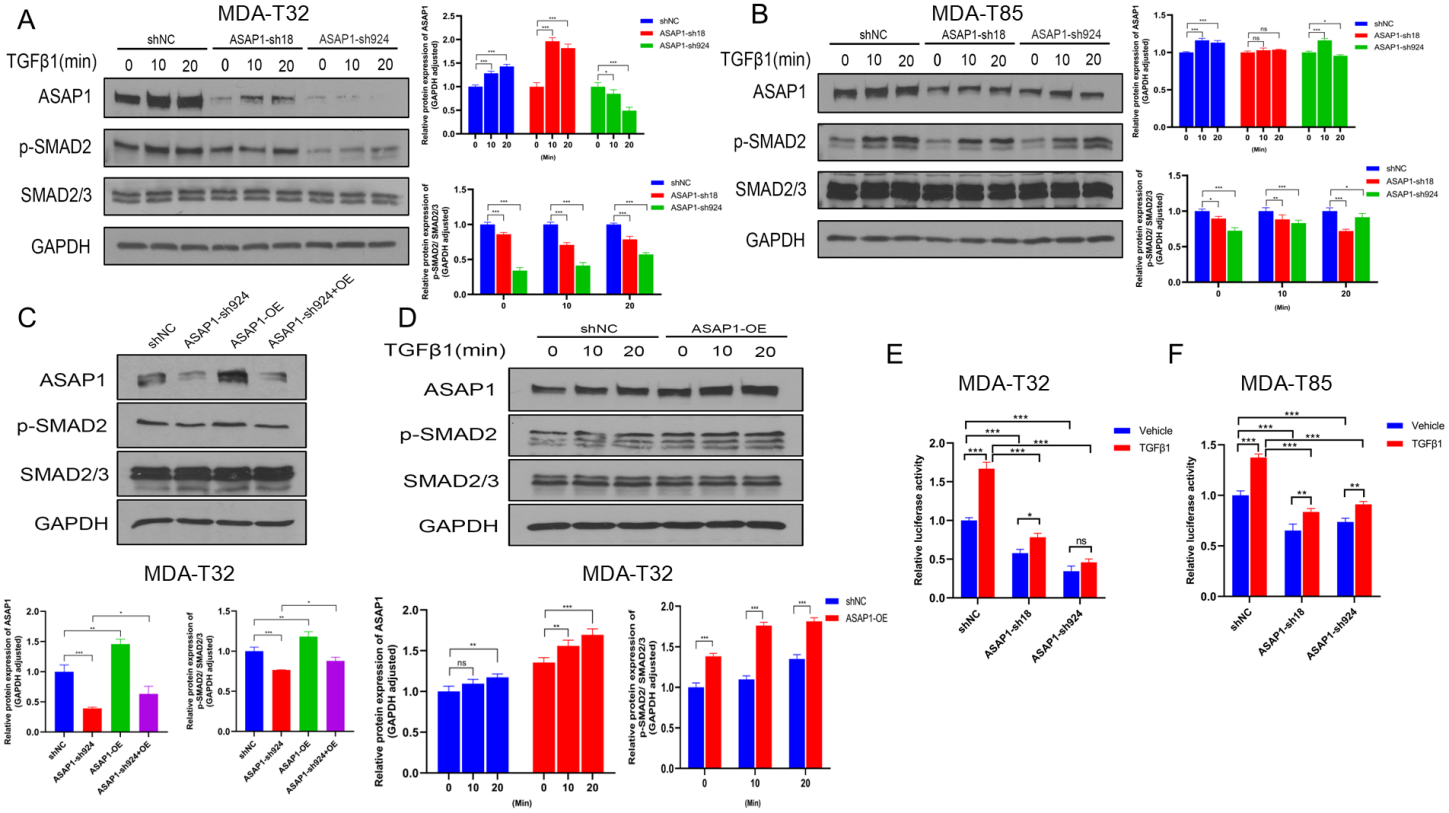
Knockdown of ASAP1 inhibits TGFβ pathway in PTC cells, while overexpression of ASAP1 can reverse these effects. (A, B) MDA‐T32 and MDA‐T85 cells from control and ASAP1 knockdown groups were treated with 10 ng/mL TGFβ1 at various time points to analyze ASAP1 and p‐SMAD2 expression and quantitative analysis. (C) Expression and quantification of ASAP1 and p‐SMAD2 were analyzed in MDA‐T32 cells across the control, ASAP1 knockdown, ASAP1 overexpression, and combined knockdown/overexpression groups. (D) MDA‐T32 cells from the control and ASAP1 overexpression groups were treated with 10 ng/mL TGFβ1 at various time points to evaluate ASAP1 and p‐SMAD2 expression and quantitative analysis. (E, F) Relative luciferase activity was measured in MDA‐T32 and MDA‐T85 cells (carrying a luciferase reporter gene to overexpress SMAD2/3/4) from the control and ASAP1 knockdown groups after 24 h of treatment with 10 ng/mL TGFβ1.

Based on KEGG pathway enrichment analysis, we identified several pathways regulated by ASAP1, including the TGFβ pathway. We selected the TGFβ pathway for further investigation due to its well‐known role in promoting EMT in multiple cancers. We treated MDA‐T32 and MDA‐T85 cells with 10 ng/mL TGFβ1 for various time points and used WB to examine ASAP1 and p‐SMAD2. ASAP1 knockdown significantly reduced p‐SMAD2 levels in both cell lines, while total SMAD2/3 levels remained unchanged, indicating that ASAP1 regulates the TGFβ pathway. TGFβ1 treatment increased ASAP1 expression levels in the control groups of MDA‐T32 and MDA‐T85 cells but had a less pronounced effect in ASAP1 knockdown cells (Figure 5A,B). Overexpression of ASAP1 in MDA‐T32 cells reversed the decrease in p‐SMAD2 expression caused by ASAP1 knockdown (Figure 5C). Furthermore, ASAP1 overexpression enhanced TGFβ pathway activation (Figure 5D). To confirm the role of ASAP1 in the TGFβ signaling pathway, we transduced a viral vector carrying a luciferase reporter gene to overexpress SMAD2/3/4 into PTC cells with ASAP1 knockdown. The results showed that ASAP1 knockdown significantly reduced luciferase activity induced by TGFβ1 in both cell lines compared to controls (Figure 5E,F).

### 3.6 Activation of the TGFβ Pathway Enhances ASAP1 Expression in PTC Cells

**FIGURE 6:**
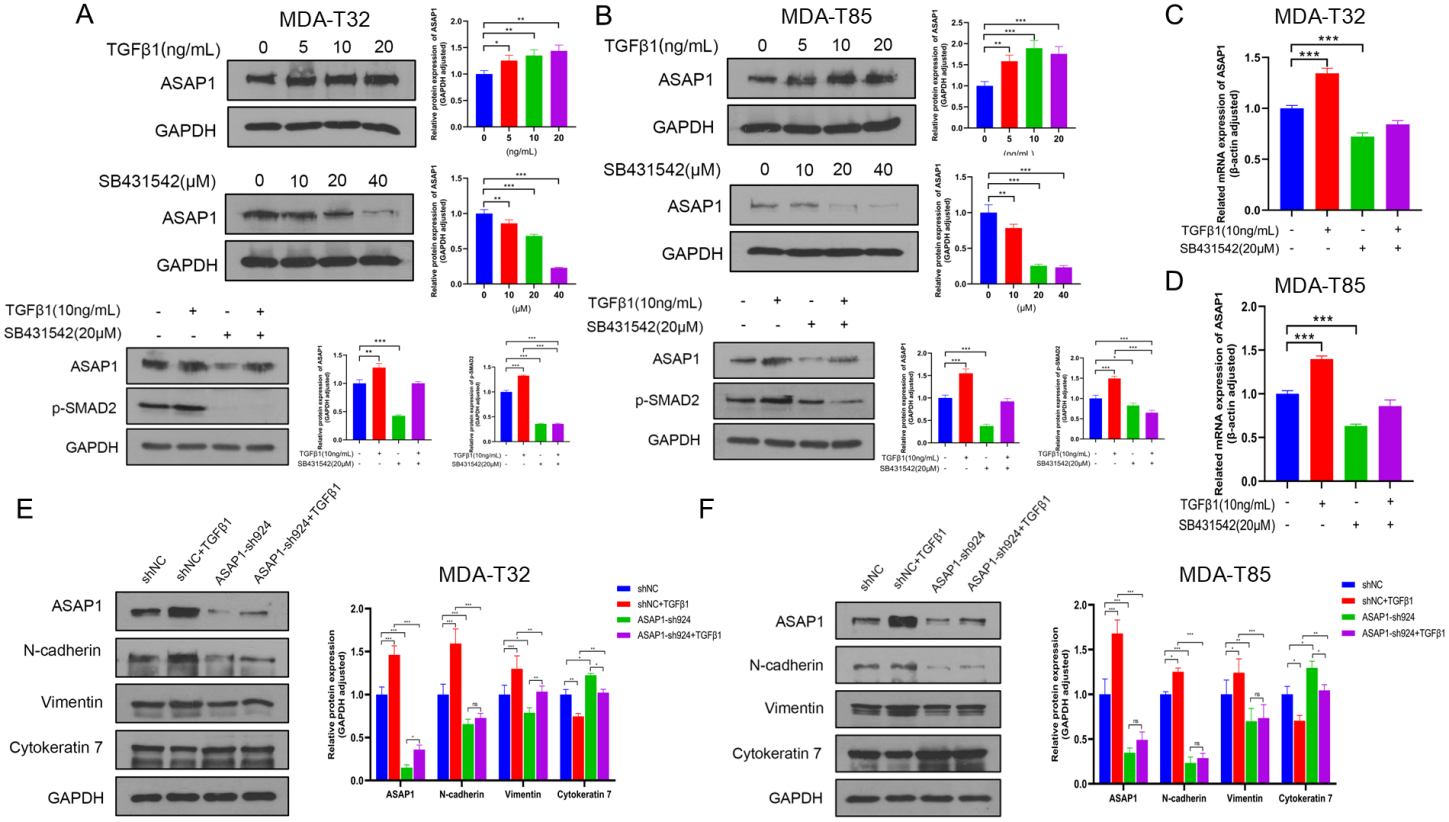
ASAP1 expression is regulated by TGFβ pathway and mediates the effects of TGFβ pathway on EMT. (A, B) MDA‐T32 and MDA‐T85 cells were treated with TGFβ1 and TGFβR1 inhibitor SB431542 for 24 h to evaluate ASAP1 protein expression. (C, D) ASAP1 mRNA expression in MDA‐T32 and MDA‐T85 cells following 24‐h treatment with TGFβ1 and the TGFβR1 inhibitor SB431542. (E, F) WB and quantitative analysis of ASAP1 and EMT markers in MDA‐T32 and MDA‐T85 cells treated with TGFβ1, comparing the control and ASAP1 knockdown groups.

To further investigate the effects of the TGFβ pathway on ASAP1 expression, MDA‐T32 and MDA‐T85 cells were treated for 24 h with increasing concentrations of TGFβ1 (0, 5, 10, 20 ng/mL) or the TGFβR1 inhibitor SB431542 (0, 10, 20, 40 μM), followed by WB to evaluate ASAP1 expression levels. We found that ASAP1 expression increased in a dose‐dependent manner with TGFβ1 treatment. Conversely, treatment with SB431542 resulted in a dose‐dependent decrease in ASAP1 expression levels. Furthermore, treatment with TGFβ1 and SB431542, either individually or in combination for 24 h, consistently regulated ASAP1 expression under both conditions (Figure 6A,B). RT‐qPCR analysis indicated that activation of the TGFβ pathway enhanced ASAP1 transcription (Figure 6C,D). In addition, TGFβ pathway activation promoted EMT in control MDA‐T32 and MDA‐T85 cells, but this effect was attenuated in ASAP1 knockdown cells (Figure 6E,F).

### 3.7 ASAP1 Interacts With SMAD2/3 and Functionally Links to the TGFβ Pathway in PTC Cells

**FIGURE 7:**
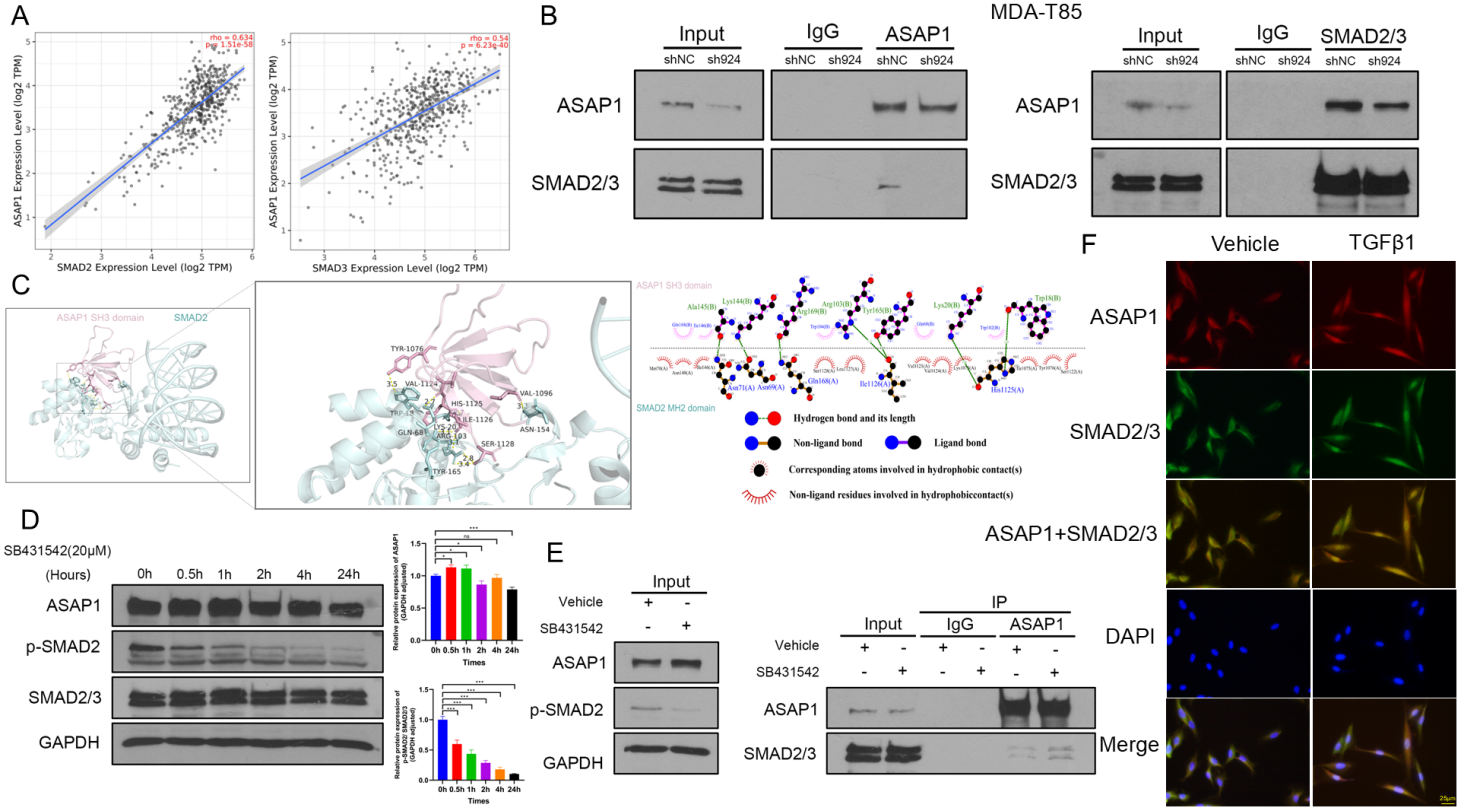
ASAP1 physically interacting with SMAD2/3. (A) Correlation analysis of ASAP1 with SMAD2 and SMAD3 in the THCA dataset using the TIMER database. (B) The interaction between ASAP1 and SMAD2/3 in MDA‐T85 cells was confirmed through Co‐IP analysis, comparing the control and ASAP1 knockdown groups. Proteins were immunoprecipitated using antibodies against ASAP1 and SMAD2/3, followed by detection via WB. (C) The potential binding sites between the ASAP1 SH3 domain and the SMAD2 MH2 domain were visualized using PyMOL and LigPlot^+^. (D) The effects of the TGFβR1 inhibitor SB431542 on ASAP1 and p‐SMAD2 expression in MDA‐T85 cells were assessed at different time points. (E) Comparison of the interaction between ASAP1 and SMAD2/3 in MDA‐T85 cells between the vehicle and SB431542‐treated groups. (F) IF was used to observe the effects of TGFβ1 on the co‐localization of ASAP1 and SMAD2/3 proteins in MDA‐T85 cells.

To investigate the potential association between ASAP1 and the TGFβ signaling pathway, we retrieved and analyzed data from the TCGA THCA cohort using the TIMER database [32]. Analysis of data from 509 patients revealed a positive correlation between ASAP1 expression and the expression of both SMAD2 and SMAD3, two key components of the TGFβ pathway, with R^2^ values of 0.634 and 0.54, respectively (Figure 7A), suggesting a potential functional relationship between them. Thus, we hypothesized that ASAP1 may interact with SMAD2/3 to regulate the TGFβ pathway. Co‐IP assays were conducted in MDA‐T85 control and ASAP1 knockdown cells using antibodies against ASAP1 or SMAD2/3. The results validated the physical interaction between ASAP1 and SMAD2/3 in PTC cells (Figure 7B). Next, we performed computational modeling to define the interaction between ASAP1 and SMAD2 using PyMOL and LigPlot+ software. We found that the SH3 domain of ASAP1 potentially binds to the MH2 domain of SMAD2, with a confidence score of 0.7975 (Figure 7C). To determine whether TGFβ pathway activity affects the interaction between ASAP1 and SMAD2/3, we treated cells with the TGFβR1 inhibitor SB431542 (20 μM) for 1 h, a time point at which TGFβ pathway activity (p‐SMAD2) was significantly suppressed while ASAP1 expression was not significantly altered. Co‐IP analysis at the 1‐h time point revealed that inhibition of TGFβ pathway activity had no significant effect on the interaction between ASAP1 and SMAD2/3 (Figure 7D,E). IF staining was used to observe the co‐localization of ASAP1 and SMAD2/3 in MDA‐T85 cells. Following TGFβ1 treatment, both ASAP1 and SMAD2/3 translocated from the cytoplasm to the nucleus, with ASAP1 expression levels being higher than those in the vehicle group (Figure 7F). These findings confirm that ASAP1 links to the TGFβ pathway through its interaction with SMAD2/3 and may be involved in its positive feedback regulatory mechanism.

## 4 Discussion

In this study, we demonstrate that ASAP1 functions as an oncogene in PTC cells by promoting EMT through activating the TGFβ pathway, suggesting its potential role in PTC metastasis. Our findings are consistent with the previous finding that ASAP1 regulates the EMT process in cancers [21, 25, 26]. Therefore, targeting ASAP1 could represent a novel therapeutic strategy for aggressive PTC by intervening in EMT‐associated metastasis.

The TGFβ signaling pathway is closely associated with tumor progression and metastasis by promoting EMT in multiple cancers [33, 34, 35, 36]. Here we found that knocking down ASAP1 attenuates the TGFβ pathway, whereas ASAP1 overexpression rescues this pathway. Therefore, ASAP1 contributes to EMT at least through the TGFβ pathway. Furthermore, the exogenous addition of TGFβ1 increases the protein expression of ASAP1, suggesting the existence of a potential positive feedback loop between the two. This further underscores the crucial interdependence of ASAP1 and the TGFβ pathway in the EMT process of PTC. Although SB43152 fully blocks SMAD activation, ASAP1 is only partially inhibited by SB43152 in PTC, suggesting that ASAP1 expression may be regulated by additional signaling pathways, such as Stat3 [15], FAK and AKT [18, 37], etc.

ASAP1 contains a PH domain, a zinc finger, three ankyrin (ANK) repeats, a proline‐rich region with an SH3 binding motif, eight E/DLPPKP repeat sequences, and an SH3 domain [14]. Previous studies have shown that ASAP1 interacts with various proteins to promote tumor progression [38, 39]. For example, ASAP1 interacts with the PH domain of PTK2 via its SH3 domain. In breast cancer, ASAP1 interacts with the SH3 domain of cortactin via its proline‐rich sequence and binds to paxillin via its own SH3 domain, playing a role in promoting invasion. Through clinical database analysis and Co‐IP experiments, we demonstrated that ASAP1 expression is positively correlated with SMAD2/3 and that ASAP1 physically interacts with the SMAD2/3 complex in PTC cells. Thus, we discovered a novel molecular mechanism through which ASAP1 regulates EMT via the TGFβ pathway. Furthermore, computational modeling predicted an interaction between the SH3 domain of ASAP1 and the MH2 domain of SMAD2 mediated by key amino acid residues. However, further investigation is required to determine whether these residues play a critical role in the interaction between ASAP1 and SMAD2/3. In addition, it remains unclear whether ASAP1 interacts with SMAD2/3 through other domains. We found that TGFβ1 treatment facilitates ASAP1 and SMAD2/3 translocation from cytoplasm to the nucleus, where the SMAD2/3 complex may also regulate ASAP1 expression as transcriptional factors to target the promoter of ASAP1 through interaction with other co‐transcriptional factors. Our RT‐qPCR data indicated that TGFβ1 indeed induced ASAP1 transcription.

We also demonstrated that inhibition of the TGFβ pathway using its receptor inhibitor did not affect the interaction between ASAP1 and SMAD2/3. This finding suggests two distinct mechanisms within the ASAP1/TGFβ axis: one involving TGFβ activity and the other involving ASAP1‐mediated protein interactions. Therefore, targeting ASAP1 or disrupting its interaction with the TGFβ pathway may effectively inhibit invasion and metastasis in PTC, providing a potential therapeutic target for its treatment.

This study is the first to systematically investigate the functional role of ASAP1 in PTC through database analysis and in vitro experiments, revealing its interaction with the TGFβ pathway and providing new insights into the molecular mechanisms underlying PTC progression. These findings enhance our understanding of the regulatory network of the TGFβ pathway. However, several limitations should be acknowledged. The current work is primarily based on in vitro models, and in vivo validation of ASAP1's function is still lacking. Moreover, it remains unclear whether the ASAP1/SMAD2/3 complex mediates feedback regulation through direct transcriptional control of target genes. Future studies should incorporate chromatin immunoprecipitation (ChIP) assays to determine whether these proteins directly bind to promoter regions of downstream targets. In addition, site‐directed mutagenesis to disrupt functional domains of ASAP1, combined with mechanistic validation in animal models, would help further clarify the specific molecular interactions between ASAP1 and the TGFβ pathway.

In summary, our study demonstrates that ASAP1 is a key regulator of EMT in PTC cells and indicates that it is functionally associated with the TGFβ pathway through its interaction with SMAD2/3. This study is the first to reveal an ASAP1/TGFβ axis in the regulation of EMT and PTC invasiveness. Thus, this finding offers a novel therapeutic target for treating PTC metastasis by targeting ASAP1.

## Author Contributions

**Shiji Song:** conceptualization; formal analysis; investigation; methodology; validation; visualization; writing – original draft. **Zixing Leng:** data curation. **Xinxin Zhao, Ziping Liu:** methodology. **Yongshuai Li:** resources; software. **Wenjing Zhang**, **Junming Yue, Yuxia Fan:** conceptualization; funding acquisition; project administration; supervision; writing – original draft; writing – review and editing.

## Ethics Statement

The authors have nothing to report.

## Conflicts of Interest

The authors declare no conflicts of interest.

## Supporting information


**Figure S1.** Expression of ASAP1 in MDA‐T32, MDA‐T85, 8305C, CAL‐62 cell lines.

## Data Availability

The data that support the findings of this study are available from the corresponding author upon reasonable request.
