# Supplementary figures and images for: ASAP1 Promotes Epithelial to Mesenchymal Transition by Activating the TGFβ Pathway in Papillary Thyroid Cancer
Source: Cancer Med. 2025 Jul 31;14(15):e71075. doi: 10.1002/cam4.71075 (PMC12311982; doi:10.1002/cam4.71075)

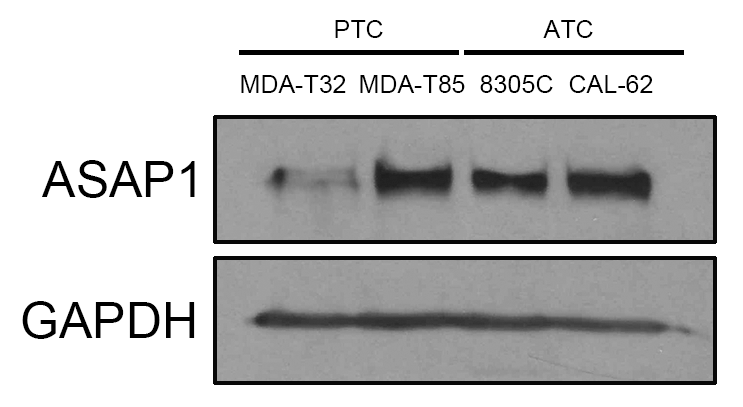

Supplement: Supplementary file 1 — Figure S1. Expression of ASAP1 in MDA‐T32, MDA‐T85, 8305C, CAL‐62 cell lines. [file CAM4-14-e71075-s001.tif]
